# Machine learning of EEG spectra classifies unconsciousness during GABAergic anesthesia

**DOI:** 10.1371/journal.pone.0246165

**Published:** 2021-05-06

**Authors:** John H. Abel, Marcus A. Badgeley, Benyamin Meschede-Krasa, Gabriel Schamberg, Indie C. Garwood, Kimaya Lecamwasam, Sourish Chakravarty, David W. Zhou, Matthew Keating, Patrick L. Purdon, Emery N. Brown

**Affiliations:** 1 Department of Anesthesiology, Critical Care and Pain Medicine, Massachusetts General Hospital, Boston, MA, United States of America; 2 Picower Institute for Learning and Memory, Massachusetts Institute of Technology, Cambridge, MA, United States of America; 3 Division of Sleep Medicine, Harvard Medical School, Boston, MA, United States of America; 4 Department of Brain and Cognitive Sciences, Massachusetts Institute of Technology, Cambridge, MA, United States of America; 5 Harvard-MIT Health Sciences and Technology, Cambridge, MA, United States of America; 6 Department of Neuroscience, Wellesley College, Wellesley, MA, United States of America; University of British Columbia, CANADA

## Abstract

In current anesthesiology practice, anesthesiologists infer the state of unconsciousness without directly monitoring the brain. Drug- and patient-specific electroencephalographic (EEG) signatures of anesthesia-induced unconsciousness have been identified previously. We applied machine learning approaches to construct classification models for real-time tracking of unconscious state during anesthesia-induced unconsciousness. We used cross-validation to select and train the best performing models using 33,159 2s segments of EEG data recorded from 7 healthy volunteers who received increasing infusions of propofol while responding to stimuli to directly assess unconsciousness. Cross-validated models of unconsciousness performed very well when tested on 13,929 2s EEG segments from 3 left-out volunteers collected under the same conditions (median volunteer AUCs 0.99-0.99). Models showed strong generalization when tested on a cohort of 27 surgical patients receiving solely propofol collected in a separate clinical dataset under different circumstances and using different hardware (median patient AUCs 0.95—0.98), with model predictions corresponding with actions taken by the anesthesiologist during the cases. Performance was also strong for 17 patients receiving sevoflurane (alone or in addition to propofol) (median AUCs 0.88—0.92). These results indicate that EEG spectral features can predict unconsciousness, even when tested on a different anesthetic that acts with a similar neural mechanism. With high performance predictions of unconsciousness, we can accurately monitor anesthetic state, and this approach may be used to engineer infusion pumps to intelligibly respond to patients’ neural activity.

## Introduction

Most surgery are performed under general anesthesia (GA). The reversible, drug-induced state consists of antinociception, unconsciousness, amnesia, and immobility with maintenance of physiological stability [1]. Anesthesiologists induce and maintain this state by administering combinations of intravenous and/or inhaled anesthetic, analgesic, and muscle relaxing drugs. Anesthesiologists primarily assess level of unconsciousness during surgery by monitoring a patient’s physiological signs (e.g., blood pressure, heart rate, respiratory rate, movement and perspiration).

Some anesthesiologists use EEG-based indices to track the state of unconsciousness [2, 3]. The most commonly used indices are the bispectral index (BIS) [4], the patient state index (PSI) [5], Wavelet-based Anesthetic Value for Central Nervous System (WAVCNS) [6], and Narcotrend [7]. Each index is computed in near real time using an algorithm that scales the output between 0 and 100, so that 100 is wide awake and 0 is profoundly unconscious. For a given index, it is assumed that same index value indicates the same anesthetic state independent of the agents being administered. Each index provides a range which the anesthesiologist is advised to target to ensure that the patient is appropriately unconscious. The range for the BIS monitor is 40 to 60, whereas the range for the PSI is 25 to 50. While these indices have helped guide anesthetic management, they can be inaccurate. For example, it is widely appreciated that the BIS index assessments of the level of unconsciousness can be when the anesthetics being used are nitrous oxide, ketamine and dexmedetomidine [8, 9]. The BIS index can also give inaccurate readings when monitoring the brain states of children under general anesthesia. To complicate things further, there are known differences in prediction of sedation/unconsciousness between these monitors [10]. Perhaps unsurprisingly, studies have been inconclusive regarding whether the use of BIS improves patient outcomes [11–13].

The reasons for these inaccuracies are now understood. A unidimensional index cannot accurately describe a patient’s state of unconsciousness for all anesthetics because the EEG dynamics of anesthetized patients change systematically with anesthetic class and mechanism of action, anesthetic dose, and patient age [14, 15]. Although propofol is an intravenous anesthetic and sevoflurane is an inhaled ether, their primary mechanism of action is through control of neural circuits in the brain and central nervous system by binding to GABA-A receptors on inhibitory interneurons [1, 15]. Not surprisingly, the EEG patterns of these two anesthetics are similar. In contrast, the presence of slow-delta oscillations in a patient anesthetized with propofol indicates a profound state of unconsciousness whereas the same oscillations produced by dexmedetomidine only suggests sedation. The reason is clear in that dexmedetomidine acts in the brain’s circuits controlled by alpha-2 adrenergic receptors and therefore, produces slow-delta oscillations through a mechanism entirely different from propofol’s GABAergic mechanism [15]. Likewise, slow-delta oscillation is thought to be due to N-methyl-d-aspartate receptor (NMDAR) antagonism in ketamine [16]. The EEG patterns of children from 1 to 18 years of age are similar to those of adults 18 to 35 years old. However, the distribution of power across the spectral bands differs [17]. In young adults, propofol’s alpha band ranges from approximately 8 to 12 Hz, whereas the corresponding band in young children ranges from 10 to 20 Hz. Power in the upper range of the 10 to 20 Hz band suggests a sedative state in an adult whereas it is characteristic of the unconscious state in a child. In adults over the age of 60, the alpha band power is likely to lie in the lower part of the 8 to 12 Hz range and have a lower amplitude relative to a young adult [14].

Recently, machine learning (ML) and deep learning (DL) techniques have been applied to pattern recognition tasks in medicine with performances similar to human interpretations [18–20], and may even improve upon human prediction of adverse events during anesthesia [21]. Previous studies have applied a wide range ML/DL features and models to predict patient unconsciousness in Intensive Care Units [22], during dosing of anesthetics to healthy volunteers [23], or in the operating room [24–26]. These studies showed that many methods from machine learning applied to raw or processed EEG data perform well in tracking Modified Observer’s Assessment of Alertness/Sedation (MOAA/S) scores or whether a patient had received an anesthetic bolus. We sought to compare the use of several different features and methods for classification and improve upon existing methods in two manners. First, we considered the explicit time-dependent nature of unconsciousness in a statistically principled fashion with Hidden Markov Models (HMMs), which can combine ML or DL EEG features from individual epochs with information on how the EEG temporally evolves between epochs and thus may yield improvements in ML classification of unconsciousness without loss of interpretability. Second, we hypothesized that incorporating known neurophysiological mechanisms by training and testing within a class of anesthetics that function via similar mechanisms would yield strong performance.

To test this hypothesis, we developed an ML training-testing-generalizability paradigm. The training cohort consisted of non-surgical healthy volunteers receiving computer-controlled infusions of propofol for approximately 2.5 hours. We recorded continuously 64-leads of EEG and computed spectral features to predict level of unconsciousness. We assessed level of unconsciousness by recording every 4 seconds the response to a binary task. We tested the classification model using a subset of the non-surgical volunteers whose data were not included in the training set. We also tested the classification model by using it on a cohort of surgical patients who received only intravenous propofol as anesthetic for induction and maintenance of unconsciousness. Their EEG activity was recorded using a 4-lead system and level of consciousness was based on the anesthesiologists clinical assessment. We finally assessed generalizability by using the classification model on a second cohort of surgical patients who received sevoflurane as their primary anesthetic for maintenance of unconsciousness.

## Results

### Study cohorts

**Fig 1:**
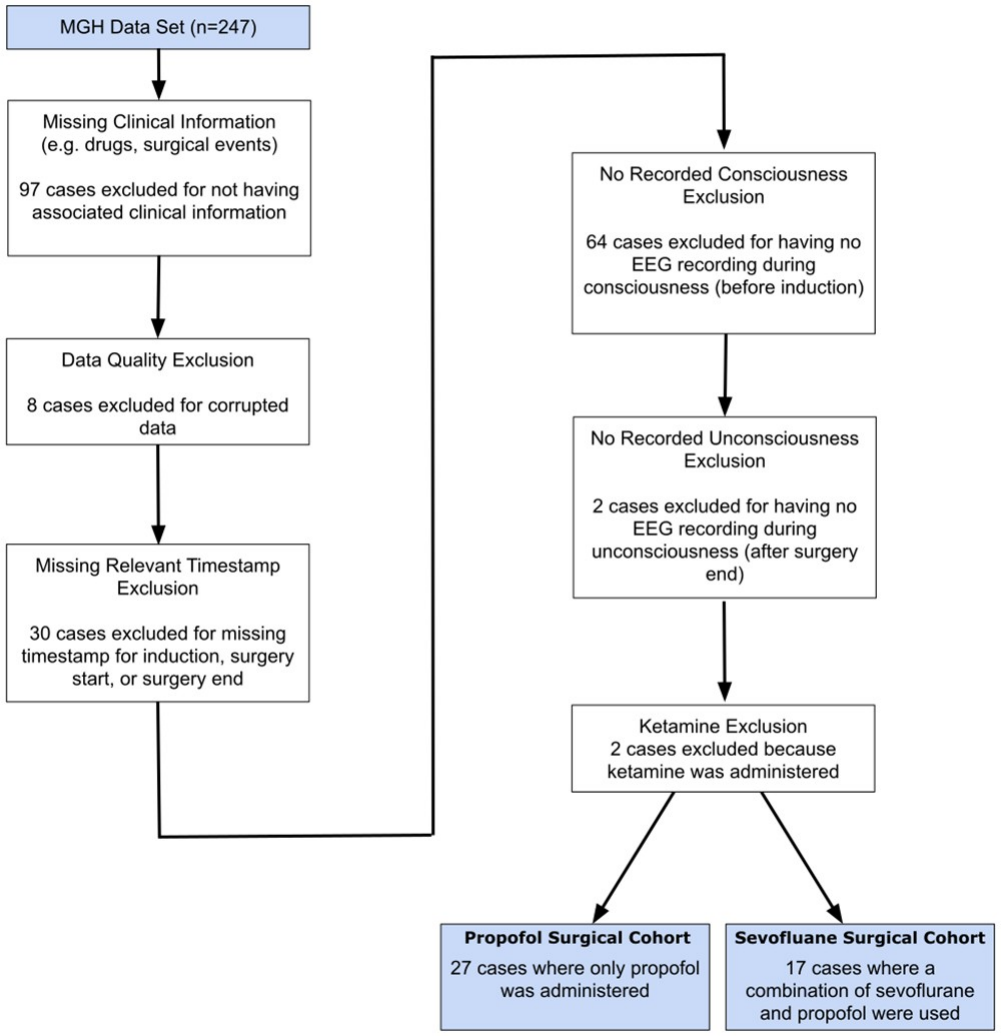
Inclusion criteria for surgical cohorts. The 44 cases included in the surgical cohorts consisted of 27 and 17 cases respectively for which propofol and sevoflurane were the primary anesthetics administered to maintain unconsciousness.

**Fig 2:**
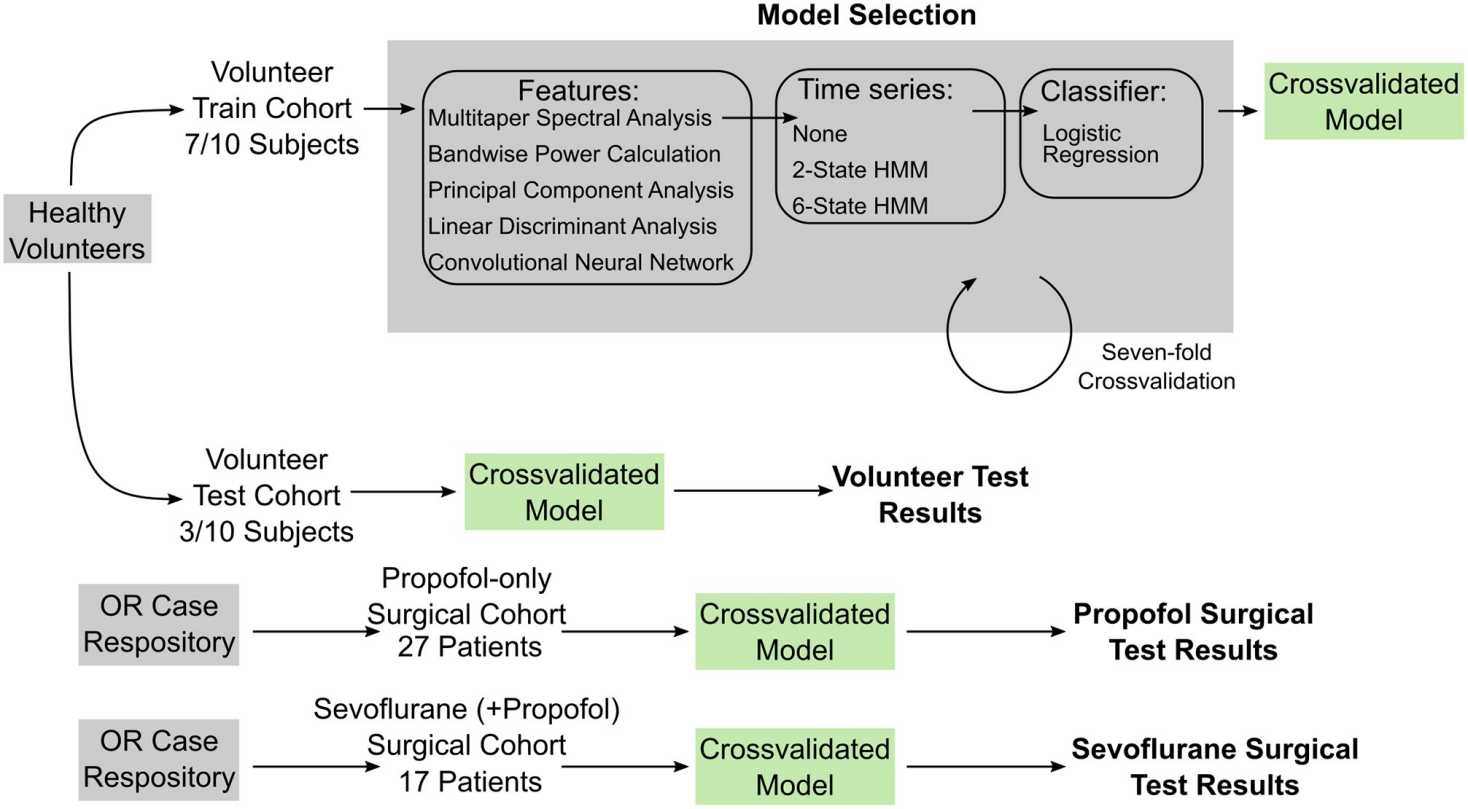
Summary schematic of machine learning approach. The healthy volunteer cohort was split into volunteer train and volunteer test cohorts comprised of seven and three subjects, respectively. Each model consisted of a feature set, a two-state or six-state HMM or no HMM, and a logistic regression classifier of the resulting time series of features. We performed model selection via sevenfold leave-one-out cross-validation on the volunteer training cohort. We selected three of the fifteen possible models and fit the models using the full seven training subjects. We applied the resulting models to data recorded from the three held-out subjects in the volunteer test cohort, the 27 patients in the propofol surgical cohort, and 17 patients in the sevoflurane surgical cohort.

We trained the ML models on data recorded from healthy volunteers administered propofol using a target-controlled infusion (TCI) protocol [27]. We tested and assessed the generalizability of the ML models using data from a hold-out set of the healthy volunteers who underwent the same TCI protocol and on data recorded from a separate cohort of surgical patients. The characteristics of the healthy volunteer and surgical cohorts are shown in Table 1, and the selection criteria for the surgical cohorts are shown in Fig 1. To approximate the relationship between unconsciousness and EEG-induced anesthetic dynamics, we re-analyzed for the healthy volunteers the time series of EEG measurements and simultaneously recorded binary (yes-no) responses to an auditory task executed every 4 seconds. We operationally defined unconsciousness as loss of responsiveness. The binary responses analyzed with a binary smoothing algorithm were used to label every 2-second interval as responsive (conscious) or unresponsive (unconscious) as in [27]. We divided the data from the 10 healthy volunteers into a training cohort of 7 subjects and a test cohort of 3 subjects. Details on the cohort are included in the Methods. A schematic of the cross-validation, model selection and testing approach is shown in Fig 2.

**Table 1:** Cohort characteristics for the healthy volunteer subjects from Purdon *et al.* 2013 and the cases included from the surgical case respository.

Statistic	Volunteer Cohort	Sevoflurane Surgical Cohort	Propofol Surgical Cohort
No. of patients	10	17	27
**Time series**			
Duration of EEG (min), mean (sd)	157 (7)	108 (67)	52 (42)
Conscious duration (min), mean (sd)	79 (16)	10 (13)	6 (13)
Unconscious duration (min), mean (sd)	78 (19)	81 (63)	38 (34)
**Patient**			
Age, mean (sd)	24 (3)	50 (20)	32 (22)
Female Sex, % (No.)	50 (5)	71 (12)	56 (15)
Height Cm, mean (sd)	171 (8)	165 (9)	167 (13)
Weight kg, mean (sd)	68.590 (8.350)	72.534 (26.938)	67.657 (17.771)
**Acuity**			
Asa 1, % (No.)	100 (10)	24 (4)	30 (8)
Asa 2, % (No.)	0 (0)	53 (9)	63 (17)
Asa 3, % (No.)	0 (0)	24 (4)	7 (2)
**Airway**			
Endotracheal Tube, % (No.)	0 (0)	76 (13)	4 (1)
Laryngeal Mask Airway, % (No.)	0 (0)	12 (2)	7 (2)
Mask Ventilation, % (No.)	0 (0)	6 (1)	4 (1)
Spontaneous Ventilation, % (No.)	100 (10)	6 (1)	85 (23)
**Procedure**			
Breast Tissue Expander Placement, % (No.)	NA	6 (1)	0 (0)
C Palate Repair, % (No.)	NA	6 (1)	0 (0)
Colonoscopy, % (No.)	NA	6 (1)	4 (1)
Colonoscopy+Egd, % (No.)	NA	0 (0)	30 (8)
D C Hysteroscopy, % (No.)	NA	6 (1)	0 (0)
Derm Surg, % (No.)	NA	0 (0)	7 (2)
Egd, % (No.)	NA	0 (0)	22 (6)
Eswl, % (No.)	NA	0 (0)	7 (2)
Exam Under Anesthesia, % (No.)	NA	6 (1)	7 (2)
Hernia Repair, % (No.)	NA	0 (0)	11 (3)
Kidney Transplant, % (No.)	NA	6 (1)	0 (0)
Laparoscopic Inguinal Hernia Repair, % (No.)	NA	6 (1)	0 (0)
Laparoscopic Rouxeny Gastric Bypass Moderate, % (No.)	NA	6 (1)	0 (0)
Laprascopic Cholecystectomy, % (No.)	NA	6 (1)	0 (0)
Laser Lithotripsy Ureteroscopy Retrograde Stent Cystoscopy, % (No.)	NA	12 (2)	0 (0)
Mastectomy With Sentinel Node Biopsy With Implant Reconstruction, % (No.)	NA	6 (1)	0 (0)
Muscle Biopsy, % (No.)	NA	0 (0)	7 (2)
Photo Selective Vaporization Of The Prostate Pvp Medium, % (No.)	NA	6 (1)	0 (0)
Scaphoid Wrist Orif Perilunate Orif And Left Carpal Tunnel Release, % (No.)	NA	0 (0)	4 (1)
Tibial Plateau Fracture Orif, % (No.)	NA	6 (1)	0 (0)
Vaginal Hysterectomy, % (No.)	NA	6 (1)	0 (0)
Surgery Vulva Biopsy, % (No.)	NA	6 (1)	0 (0)

### Cross-validation and model selection

**Fig 3:**
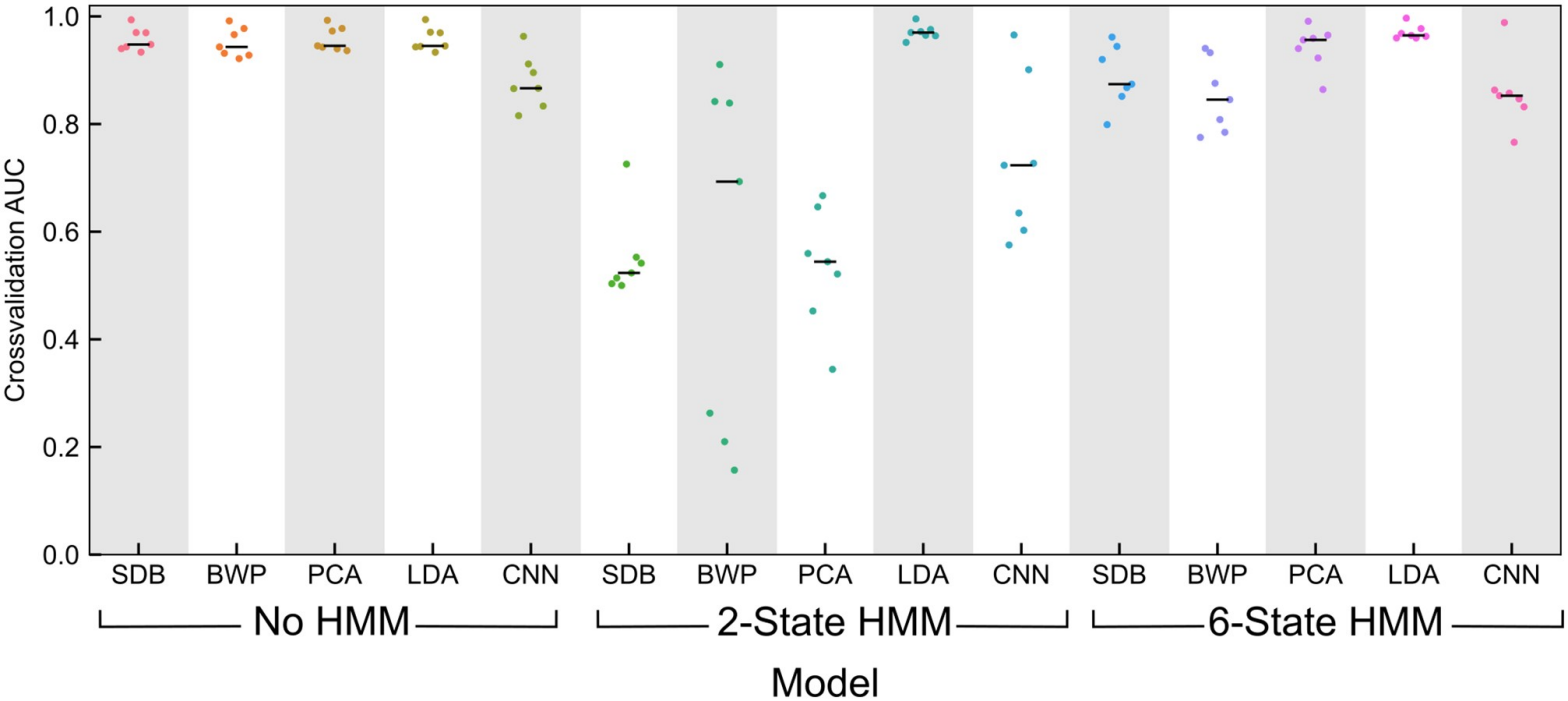
Cross-validation and model selection results. Area under the receiver operating characteristic (ROC) cuve (AUC) on the left-out cross-validation subjects for the 15 candidate models. A model consisted of one feature set (multitaper spectrogram: SDB, bandwise power: BWP, principal components of the spectrogram: PCA, linear discriminant scores: LDA, principal components of features generated by a convolutional neural network: CNN) and one time series treatment (no treatment of features as a time series, a two-state HMM: HMM2, a six-state HMM: HMM6). Median AUC is shown with a black bar. We chose to keep three models with similar performance to examine in detail (shown in bold). These models were LDA+HMM2 (best performance and more parsimonious than LDA+HMM6), PCA with no treatment of features as a time series (best non-HMM performance, used in control previously [28]), and SDB with no treatment of features as a time series (simplest case).

We used seven-fold cross-validation to estimate how trained models will perform on unobserved patients. We used cross-validation results to select 3/15 models that performed well and yield clinically relevant interpretations. We trained these models using the full volunteer train cohort (7 subjects) and applied the resulting models to the volunteer test cohort and surgical test cohorts. Cross-validation and model selection results are shown in Fig 3. We found that many models performed well (mean AUC > 0.9 on the left-out subject from each of the seven folds) during cross-validation.

We chose three models with similarly high performance, as shown in bold on Fig 3: mutlitaper power spectrum in decibels (SDB) features with no HMM, principal component analysis (PCA) features with no HMM, and linear discriminant analysis (LDA) features with a two-state HMM (LDA+HMM2). Details of the featurization and HMM is provided in the Methods. These models yielded mean AUC scores of 0.957, 0.958, and 0.970, respectively, for the left-out subject during cross-validation. LDA with a six-state HMM also performed well (mean AUC = 0.970) but we considered this to be redundant with LDA+HMM2 and did not keep this model for further analysis. We did not perform significance testing for differences in AUC between models during cross-validation due to the low sample size and large number of potential models. Despite the small number of volunteers, each volunteer recording consisted of approximately 2h of EEG data separated into 2 s epochs for training, increasing the power of our approach despite the dependence of samples from the same subject.

### Model testing: Volunteer cohort

**Fig 4:**
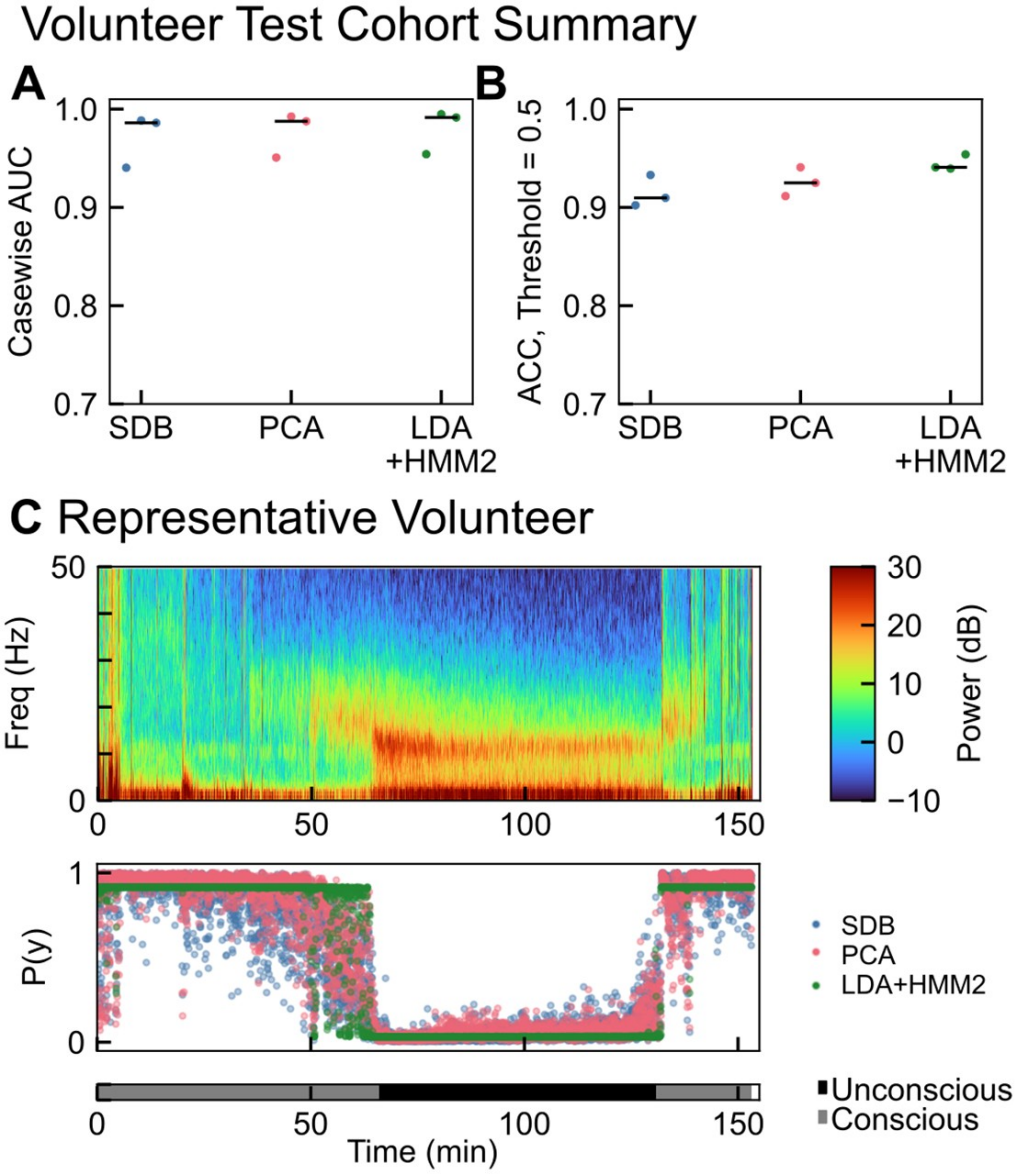
Model performance on volunteer test cohort (n = 3). (A) Casewise AUCs for each of the three selected models with median values shown in black. Median AUCs were 0.986, 0.988, and 0.991 for SDB, PCA, and LDA+HMM2 respectively. (B) Casewise accuracy for each of the three selected models with median values shown in black. Median accuracies were 0.910, 0.925, and 0.941 for SDB, PCA, and LDA+HMM2 respectively. (C) Model performance on a representative subject, showing the multitaper spectrogram and model-predicted probability of consciousness (P(y)) and ground-truth conscious state as recorded in [27].

We tested the selected models first on EEG recordings from three healthy volunteers in the same study used for training [27]. As shown in Fig 4A, the SDB, PCA, and LDA+HMM2 models yielded median AUCs of 0.986, 0.988, and 0.991 respectively for the held-out volunteers. Accuracy was similarly high, with medians of 0.910, 0.925, and 0.941 for each model, using the default threshold of 0.5. A representative subject is shown in Fig 4C. Notably, after propofol was administered but before the subject lost consciousness, the SDB and PCA models show a progressive decrease in the probability of consciousness. In contrast, the LDA+HMM2 model shows an abrupt transition between conscious and unconscious. While the LDA+HMM2 model yielded a slightly higher AUC, it did so at the expense of continuous probability of consciousness values.

### Model testing: Propofol surgical cohort

**Fig 5:**
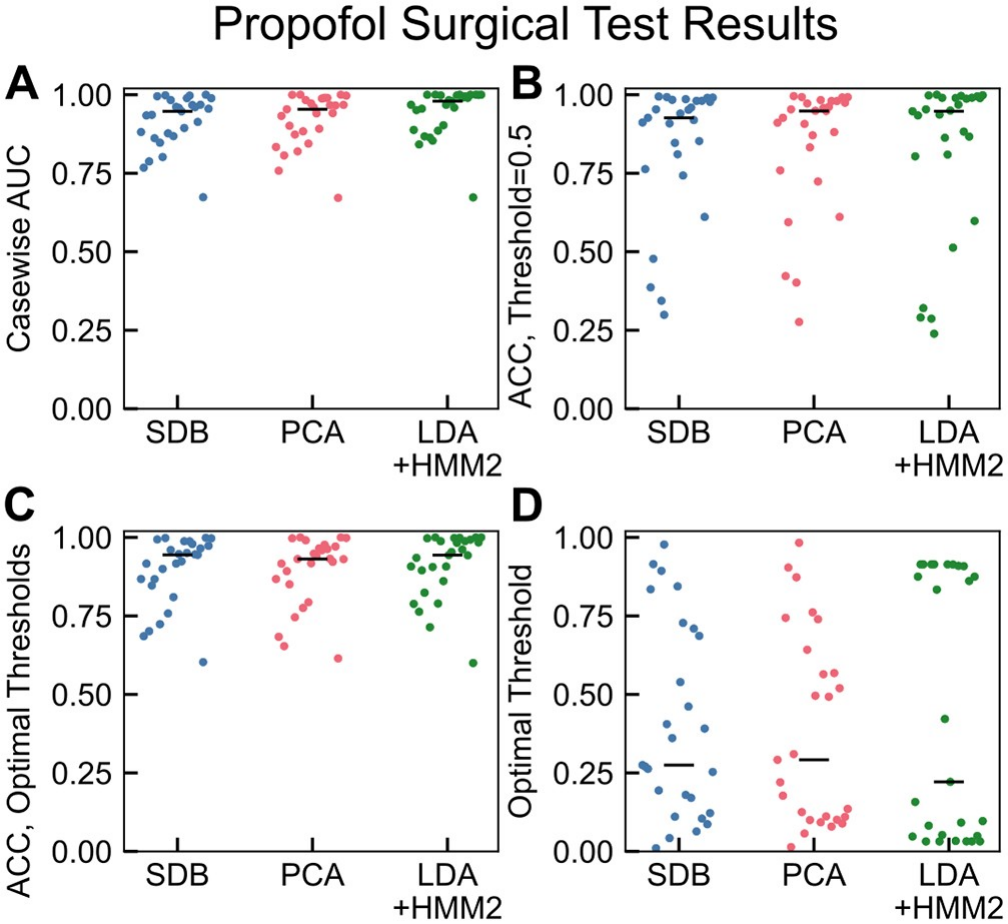
Model performance on propofol surgical test cohort (n = 27). (A) The classifier model performances were high on a by-case basis, with median casewise AUCs of 0.947, 0.953, and 0.980 for SDB, PCA, and LDA+HMM2 respectively. The lowest-performing case was consistent between classifiers and is examined further in S1 Fig. (B) Classifier accuracy with default threshold of 0.5 was high on average (median accuracies 0.926, 0.948, and 0.948 for SDB, PCA, and LDA+HMM2 respectively). However, several cases had low accuracy, likely due to improper inter-individuality in optimal threshold. (C) Although median accuracy is nearly identical for case-specific thresholds (0.944, 0.931, and 0.944 for SDB, PCA, and LDA+HMM2 respectively), performance for low-accuracy cases improved. (D) Optimal threshold was determined by maximization of (1 − *FPR*) + *TPR*, as described in methods. A threshold personalization procedure would be essential for clinical accuracy.

Next, we applied the three classification models trained on healthy volunteers to data collected during surgical cases that used a propofol total intravenous anesthesia approach (TIVA) for GA. That is, propofol was used for both induction and maintenance of unconsciousness. The healthy volunteer EEG recordings were collected in a tightly controlled environment. In contrast, the surgical data were measurements during surgery, used different EEG equipment, and were recorded from patients with a wider range of age and health status (see Table 1 and Methods for details). We designated EEG data collected prior to induction and after surgery started as conscious and unconscious, respectively, and made no predictions between induction and surgery start. Despite the data collection differences, the classification models generalized well with median casewise AUCs of 0.947, 0.953, and 0.980 for SDB, PCA, and LDA+HMM2 respectively (Fig 5). These results are comparable to those found for propofol in [23, 24]. Although median accuracy was also high with a default threshold of 0.5, some cases exhibited low accuracy (<0.50). Tuning the individual threshold by maximizing true positive rate (TPR) and minimizing false positive rate (FPR) improved the accuracy for the least-accurate cases. Selecting a personalized classification threshold may be useful in a surgical context and may improve performance. We also note that accuracy here is affected by the imbalance between conscious and unconscious duration for each patient.

**Fig 6:**
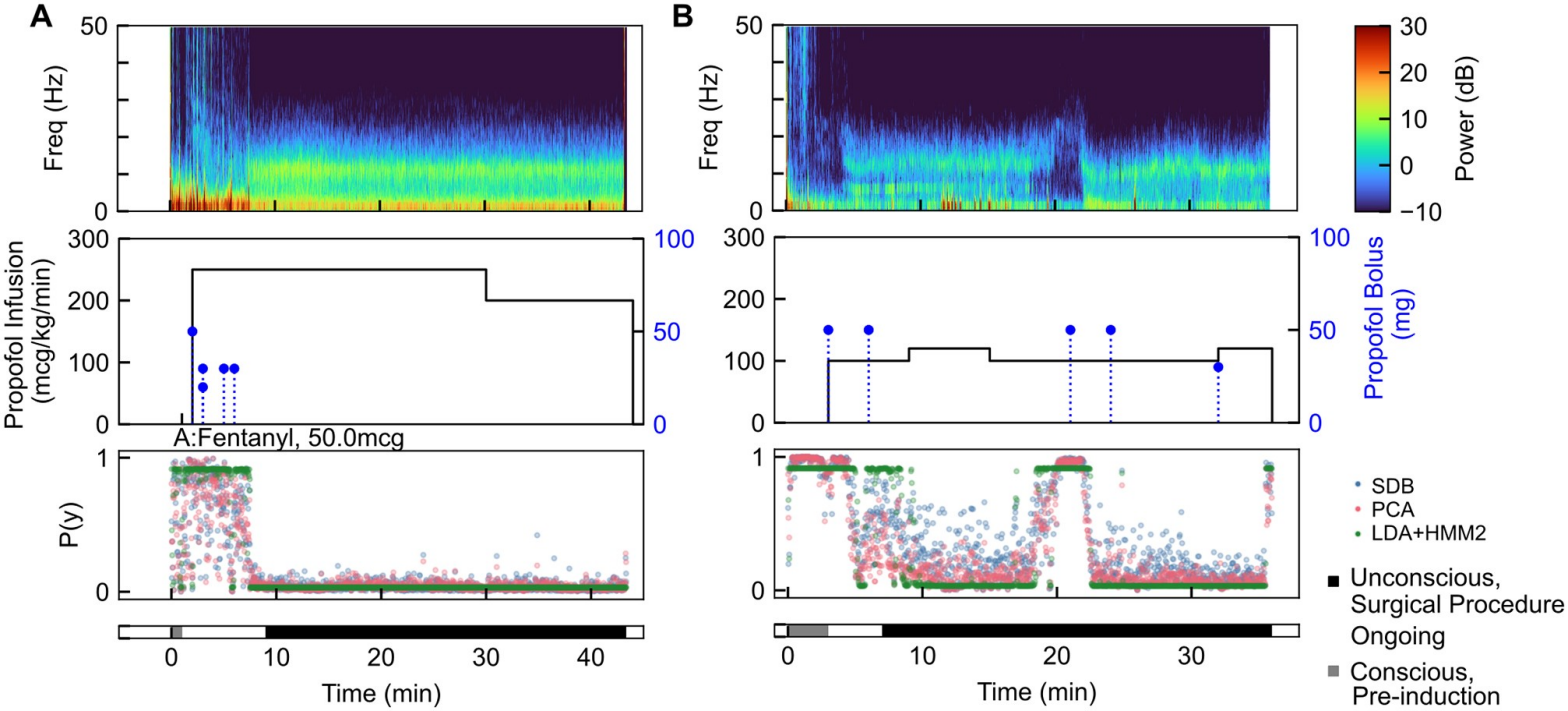
Two example propofol surgical cohort cases with multitaper spectrogram of EEG data (A,D), propofol and other drug doses (B,E) and model-predicted probability of consciousness (C, F) with patient state as determined by clinical records. (A-C) Following a brief pre-induction conscious epoch, the patient was induced using a bolus of propofol. The procedure (black bar, bottom) started after the final induction bolus, and the model predictions indicated loss of consciousness only toward the end of the epoch between induction and procedure start. The model predicted that the patient remained unconscious throughout the procedure. (D-F) The patient was induced using two propofol boluses and the model predicted loss of consciousness. At approximately 19 minutes, the model begins to predict the return of consciousness. Several minutes later, the patient was administered two propofol boluses, a real-time adjustment by the anesthesiologist in response to an inadequate level of unconsciousness, which was correctly detected by the model.

We examined the propofol surgical cohort predictions in detail to determine if the models yield information that may be subjectively useful during a surgery. We tested the classification model and found that for an example volunteer and an example surgical case, performing prediction for each 2 s epoch took <0.1s on a MacBook Pro using a 2.4 GHz Quad-Core Intel®Core i5 with 16 GB RAM. This indicates potential for the algorithm to be run in real-time without unreasonable computational need (S3 Fig). Fig 6 shows two representative cases for application of the classification models to data from the OR.

The first case (Fig 6A) was a 28-year-old man undergoing a colonoscopy. The patient received five propofol boluses in the ten minutes preceding the procedure start and an infusion of propofol throughout the procedure. The classifier models correctly predicted consciousness during the short pre-induction epoch, and continued to predict that the patient was responsive until just before the start of the procedure (black bar, bottom). The classifier continued to predict an unconscious state for the duration of the procedure, and likewise the anesthesiologist did not add any additional propofol boluses after the procedure began.

The second case (Fig 6B) was a 44 year old woman undergoing a left nipple inversion correction. In this case, the patient received two bolus doses of propofol prior to the start of the procedure start and unconsciousness was maintained by an infusion of propofol. The models correctly predicted consciousness pre-induction, loss of consciousness during the induction period, and a deeply sedated state during the start of the surgery. Just before 20 min post-EEG start, all three classifier models began to predict a higher probability of the patient being consciousness. At approximately minute 19 and following the model prediction of recovery of consciousness, the anesthesiologist administered two additional propofol boluses, and the models correctly predicted that the patient returns to a state of unconsciousness. Here, the models may have correctly identified a situation of inadequate sedation during a case, showing subjectively that such an approach may be useful in the OR. This is especially suggestive because the tracking algorithm predicted the return to consciousness several minutes before the anesthesiologist administered additional propofol boluses. A third case (with the lowest casewise AUC) is shown in S1 Fig. Here we note that the label assigned retrospectively may not correspond well with the true patient state during surgery; it appears that the patient was indeed minimally conscious and sedated until more than ten minutes following the start of the procedure.

### Model testing: Sevoflurane surgical cohort

**Fig 7:**
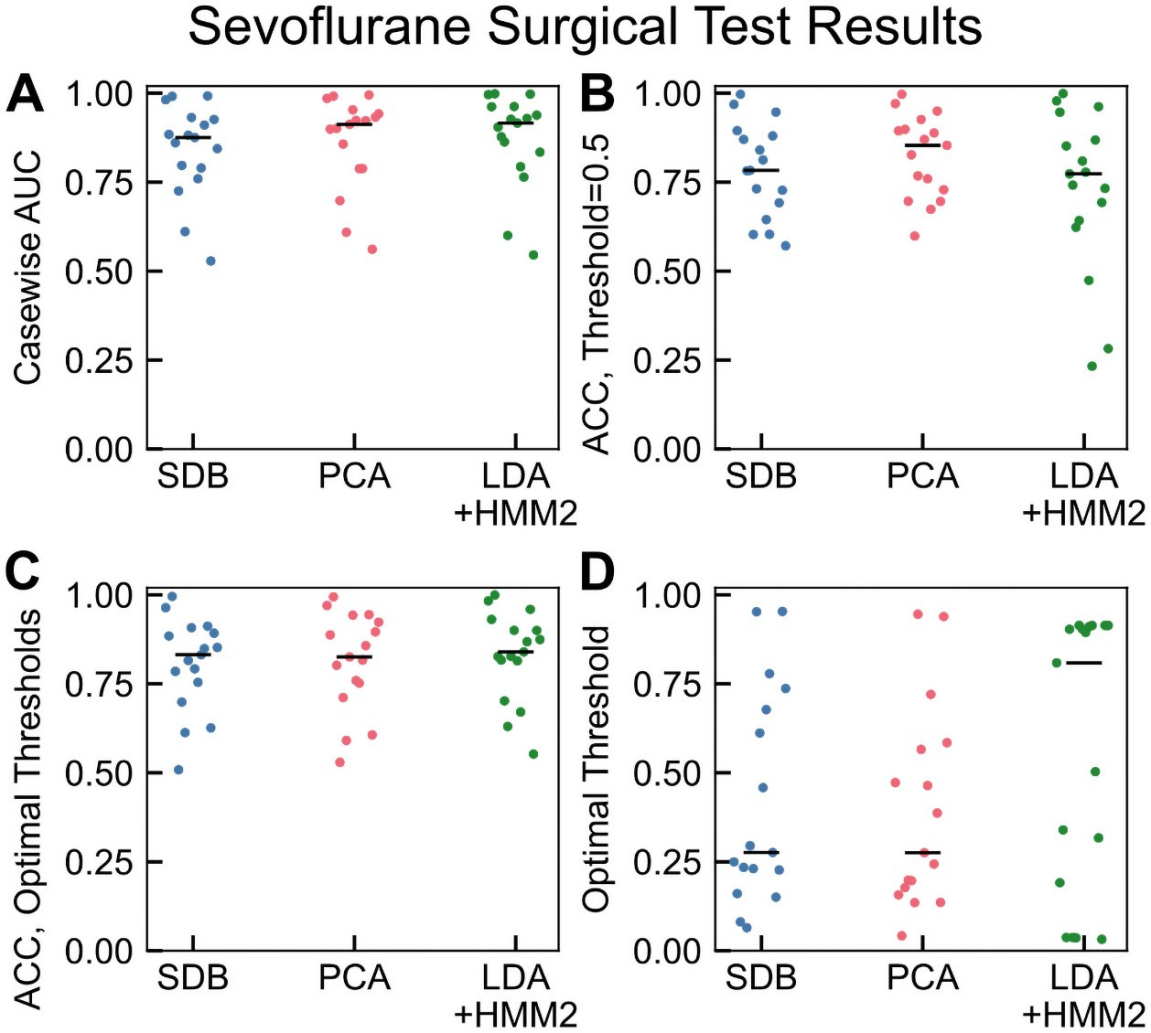
Model performance on sevoflurane surgical cohort (n = 17). (A) The classifier model performances remained high on a case-by-case basis, with median casewise AUCs of 0.875, 0.912, and 0.916 for SDB, PCA, and LDA+HMM2 respectively. (B) Classifier accuracy with default threshold of 0.5 was moderate on average (median accuracies 0.783, 0.853, and 0.773 for SDB, PCA, and LDA+HMM2 respectively). (C) As with propofol, case-specific thresholds for sevoflurane yielded improvements for poorly performing cases but little change on average (median accuracies 0.832, 0.825, 0.840 for SDB, PCA, and LDA+HMM2 respectively), performance for low-accuracy cases improved. (D) Optimal threshold was determined by maximization of (1 − *FPR*) + *TPR*, as described in the Methods.

Data recorded during an additional 17 cases were identified where sevoflurane was used to maintain unconsciousness as shown in Fig 7. Although sevoflurane EEG recordings during general anesthesia differ slightly from propofol alone, we expected moderate generalizability because both propofol and sevoflurane both alter neural circuit activity by binding to GABA receptors. We found that our models performed well for the sevoflurane cases, with median casewise AUCs of 0.875, 0.912, and 0.916 for SDB, PCA, and LDA+HMM2 respectively. Our AUCs in this test dataset were comparable to the two sevoflurane cases in [24] and shown for a larger cohort. Meanwhile, these AUC scores improve on performance in comparison to sevoflurane cases in [23]. Still, accuracy using a default threshold of 0.5 was in some cases low, indicating that these classification thresholds must be tuned.

## Discussion

Automated tracking of unconsciousness during clinical anesthesia has the potential to enable precise titration of anesthetics and can be used readily in conjunction with current anesthesia practice. Here, we sought to develop classification models for making a binary conscious/unconscious decision using 2s epochs of EEG. Most classification models performed remarkably well given the straightforward nature of our approach and the simplicity of using logistic regression for classification. The high AUC by case (>0.95 for propofol cases) indicated that more complicated machine learning approaches are unnecessary for tracking conscious state during anesthesia. This is due to the clear and large-magnitude difference in signatures of consciousness or unconsciousness in the recordings, as well as the use of a gold-standard dataset for training. While anesthesiologists have read EEG to monitor the brain clinically, our result supports this approach. This study extends previous works which indicated that signatures of consciousness differed from those of unconsciousness during anesthesia by showing that the signatures of (un)consciousness are sufficient to discriminate between states with a high performance. Importantly, the model performance remained high when applied to cases recorded during GA for surgery, and when using the GABAergic anesthetic sevoflurane. Generalizability across a patients undergoing anesthesia maintained by GABAergic anesthetics points the potential to use known physiology to construct or apply classification models.

Classification models trained using PCA and SDB performed comparably, indicating that the information pertinent to making the classification is maintained through the linear PCA dimensionality reduction. The use of an HMM did not show improvement in AUC for features generated in an unsupervised fashion. This is likely because HMM-learned hidden states are unsupervised and did not correspond well with conscious/unconscious states, thus adding temporal stability with an HMM was unable to improve prediction. When LDA was used to generate features in a supervised fashion, the HMM was able to add temporal continuity to the supervised features and thus performance exceeded our other models. The benefit of using an HMM with LDA is incorporating the inherent temporal continuity of the state of consciousness: a patient is unlikely to flip rapidly between states of consciousness, and the HMM provides some increased robustness to noisy flipping between states. The cost of this temporal continuity is that the binary state of the HMM results in a more discontinuous transition between likely-conscious and likely-unconscious states (as shown by the less smooth transitions in in Figs 4 and 6). Approaches for closed-loop anesthesia delivery (CLAD) may find a marker of conscious state with more dynamic range, such as the PCA or SDB classifier a more suitable signal for control. Although the use of deep learning in the form of convolutional neural network featurization did not yield useful results in this study, we cannot rule out that deep learning may provide utility in a future study. We also emphasize that spectral features are not exhaustive: any number of features, from raw EEG signal to Granger causality to any number of hand-picked features may be used for classification. We elected to use spectral features alone due to a well-established link between EEG spectral composition, anesthetic dose, and unconsciousness [1–3, 14]. A training set that is larger by one or more orders of magnitude may yield increasingly useful features.

Our classification models performed favorably in comparison to previous studies involving the use of machine learning to track conscious state. One recent study was able to predict depth of anesthesia in one of four qualitatively defined classes (from “profound unconsciousness” to “awake”) in real time with average accuracy = 0.92 using hand-selected features [29]. The good average performance of the classifier used in this study is complicated by an unclear clinical/physiological inference of the “true” patient state, which was subjectively assigned in a post-hoc manner. A different study constructed a classification algorithm using EEG collected during sedation driven by several anesthetics and tested using similar healthy volunteers [23]. In comparison to this study, our models for tracking propofol unconsciousness performed comparably (mean AUC > 0.9), however, our models outperformed the models from [23] that were applied to sevoflurane cases. Our performance on our cohort of 17 sevoflurane cases was more comparable to the two sevoflurane cases in [24]. Another recent study that sought to identify EEG patterns of emergence during sevoflurane anesthesia found four distinct patterns, and used spectral features (primarily ratio of delta to alpha power) to distinguish between these patterns [30]. While this approach performed well (mean accuracy = 0.7-0.9), it was used to retroactively classify entire emergence epochs rather than track depth of anesthesia. Our models had higher performances than a prior study predicting consciousness and delirium in Intensive Care Units (ICUs) [22]. Our higher performance may reflect the more profound level of unconsciousness required for GA than is required for sedation in the ICU, less signal noise in the more controlled operating room, or greater variability in the brain states of the severely ill ICU patients.

Beyond performance, there are several advances in this study. Prior classification models did not use continuous measures of patient responsiveness to construct models and instead relied on (frequently post-hoc or subjective) human labeling to generate a set of data for training. The successful transfer of models to a real-world setting favorably indicates that data recorded under tightly controlled study conditions indeed matched well with data recorded during clinical anesthesiology practice. Furthermore, prior consciousness monitoring studies with surgical data often handpicked clean regions of EEG data to form training and testing sets, avoiding the frequently noisy and imprecise nature of surgical recordings. Here, we applied our classifier to EEG data recorded during entire surgical cases, and found results indicating that our approach will potentially work even in the frequently hectic operating room environment. Aside from differing recording conditions, the surgical cases were recorded using different hardware (4-lead EEG in OR vs. 64-lead EEG in study of healthy volunteers) and software, further indicating that our model features are highly generalizable and do not require tuning to each specific system that may be in use.

Despite these successes, there are significant limitations to our approach. First, our training cohort is small and homogeneous in age and health status. EEG signatures of unconsciousness are known to vary with age and health [14], and so a more generalizable approach would involve training using a larger and more diverse cohort. We found that the signatures identified in healthy volunteers translated surprisingly well to the surgical test cohorts, which was notably older and sicker by ASA standards. Still, true personalization may involve finding individualized thresholds in order to attain the high casewise AUC performance values in Fig 5. Future work will seek to determine how such a threshold might be adjusted as a function of patient clinical characteristics such as age or ASA status. The propofol surgical cohort was also comprised primarily of simple and brief surgeries, whereas deeper states of unconsciousness are common during more invasive procedures such as cardiac surgeries. A cohort with greater variety in surgical conditions would also improve detection of burst suppression, a very deep state of anesthesia or medically induced coma [1] which was sometimes misclassified as consciousness, as in S4 Fig. This is because during suppression epochs EEG is isoelectric (flat), and lacks the strong slow-alpha power characteristic of GABAergic modulation necessary used to identify unconsciousness. That the classifier differentiated between unconsciousness and burst suppression/coma suggests a three-state classification model may improve brain monitoring during anesthesia, where consciousness and coma are both undesirable. However, we did not seek to include burst suppression classification in this study because existing burst suppression segmentation methods perform well [31].

Finally, we note that our tracking algorithm does not seek to replace anesthesiologists, but instead to provide additional information to assist in guiding anesthetic administration. An anesthesiologist will be able to incorporate additional information about patient status to better assess consciousness. Our results support the utility of developing algorithms for tracking unconsciousness under a supervised learning framework. Finally, our results support the development of algorithms for tracking unconsciousness in a classwise manner grouped by anesthetic mechanism.

## Methods

### Datasets

All data were collected in accordance with relevant guidelines, and approved by the Human Research Committee at Massachusetts General Hospital.

#### Healthy volunteer cohort

The healthy volunteer cohort consisted of ten subjects between the ages of 18-36, American Society of Anesthesiology Physical Status I, and with Mallampati Class I airway anatomy. Clinical details are provided in Table 1 and Ref. [27]. Propofol was administered via computer-controlled infusion to achieve target effect-site concentrations of 0, 1, 2, 3, 4, and 5 *μ*g/mL. Each target concentration was held for 14 minutes. Propofol infusion rates were decreased in a stepwise fashion so that both induction of and emergence from unconsciousness were gradual. Unconsciousness was measured by whether a patient responded to auditory stimuli. Auditory stimuli (clicks or words) were presented every 4s in a repeating sequence of click-click-word-click-click, with a total of 210 stimuli per effect-site concentration level. Subjects were instructed to press one button if they heard a word and another button if they heard a click. Stimuli details are further described in [27]. Unresponsiveness in this setting corresponded to unconsciousness because subject were unresponsive to auditory stimuli.

In order to determine the probability of response to click and verbal stimuli, Bayesian Monte Carlo methods were used to fit a state-space model to these data. Loss of consciousness (LOC) was labeled as the first time following induction at which the probability of response to an auditory stimulus (*P*_*verbal*_) was less than 0.05 and remained so for at least 5 min. Return of consciousness (ROC) was the first time during emergence at which *P*_*verbal*_ was greater than 0.05 and remained so for at least 5 min.

EEG was recorded using a 64-channel BrainVision MRI Plus system (Brain Products) with a sampling rate of 5,000 Hz, bandwidth 0.016-1000 Hz, and resolution 0.5*μ*V least significant bit. The Fp1 channel was used for all further analysis. Subjects were instructed to close their eyes for the duration of the study to avoid eye-blink artifacts in the EEG.

The healthy volunteer cohort was divided into a training cohort (seven subjects), and a test cohort (three subjects).

#### Surgical cohorts

Two cohorts were created by reviewing a database of 247 patients who underwent general anesthesia and simultaneous EEG recording collected between November 1, 2011 and August 20, 2015. Excluded from the cohort were cases without corresponding clinical information, cases with ketamine used a primary anesthetic, cases with corrupted or improperly recorded data, cases without a relevant timestamp (induction, surgery start, or surgery end), and cases without the EEG recorded both before induction and after surgery start. We excluded ketamine cases because ketamine is known to have a very different spectral signature of effect, due to its role primarily as an NMDA antagonist [32]. Ultimately, 44 cases were deemed suitable for analysis and a full breakdown is shown in Fig 1. Data collected during these cases were split into a propofol surgical cohort (where propofol alone was used for induction and maintenance of GA) and a sevoflurane surgical cohort (where sevoflurane was used for maintenance of GA though propofol was commonly used for induction).

Patient status was labeled as consciousness or unconscious using surgical timestamps of induction, surgery start, and surgery end. All EEG epochs before induction were labeled as conscious, and all epochs between surgery start and surgery end were labeled as unconscious. Times in between induction and surgery start were labeled as an undetermined state of consciousness and all times after surgery end were not used.

Frontal EEG data were recorded using the Sedline brain function monitor (Masimo Corporation, Irvine, CA, USA). The EEG data were recorded with a pre-amplifier bandwidth of 0.5-92 Hz, sampling rate of 250 Hz, and with 16-bit, 29 nV resolution. The standard Sedline Sedtrace electrode array recorded from electrodes located approximately at positions Fp1, Fp2, F7, and F8, with ground at Fpz and reference electrode 1 cm above Fpz. Impedance was less than 5 kΩ for each electrode. Only electrode Fp1 was considered in all further analyses. Epochs where EEG signal dropped out were removed by filtering out epochs with a total power less than -30,000dB.

### Signal processing

#### Detrending and preprocessing

EEG recordings from healthy volunteers were down-sampled to 250 Hz before analysis using an anti-aliasing filter. EEG data recorded from all individuals were processed in a manner enabling real-time assessment of conscious state, with no retrospective analysis requiring knowledge of the full time series in advance. EEG data were windowed for multitaper spectral analysis and detrended via a linear detrend of each window (as in [27]). We discarded epochs with very low power (<-30,000 dB), which generally indicates that the sensor was disconnected from the patient. The most prominent artifacts observed were before/during induction and at times in the middle of the surgery, which we suspect is due to the surgical and anesthesia care teams moving or repositioning the patient.

#### Multitaper spectral analysis

The power spectrum and spectrogram were computed for each subject using the multitaper spectral estimation methods implemented in the NiTime package [33]. The spectrogram is an estimate of the EEG power spectrum performed on consecutive windows of EEG data. The spectrograms of the Fp1 electrode were computed using the following parameters: window length T = 2 s with no overlap, time-half-bandwidth product TW = 3, and a spectral resolution of 3Hz. 2TW-1 = 5 tapers were used, resulting in better than 90% spectral concentration within the bandwidth [34]. An adaptive weighting routine was used to combine estimates of different tapers as described in [35]. This resulted in a set of power spectral estimates collected at 2 s intervals for the duration of an anesthetic regimen.

### Classification models

Each classification model consisted of a feature set, a model for treating the time-evolution of the features, and a logistic regression classifier to map the resulting temporal series of features to prediction of unconsciousness.

We developed classification models by processing EEG data into a feature set, adding a time series analytical approach via Hidden Markov modeling, and then training a classifier to learn a representation between EEG features and patient conscious state. The feature sets used were the multitapered EEG spectral power (denoted SDB), EEG bandwise power (denoted BWP), the first three principal component [36] scores of the multitaper spectrogram (denoted PCA), the linear discriminant [36] score of the multitaper spectrogram (denoted LDA, with supervised learning performed by including the labels), and the first ten principal component scores of a set of features generated by a deep convolutional neural network (denoted CNN). Features for each epoch were either directly passed to a logistic regression classifier [36] to map features to patient conscious state, or input into a HMM to model evolution in time. In the HMM case, the HMM state likelihood from the prior 2 s epoch was used in the logistic regression classification. The EEG features updated every 2 s and models were developed such that they may be run in real time and predict unconsciousness in real time.

#### Featurization methods

Five sets of features were derived from the EEG multitaper spectrogram. These features were:

- **The multitapered power spectral estimate (**X**^(*SDB*)^)**: Formally, this matrix of features is $S\in R^{100\times N}$ which is the multitapered power spectral estimate at each of *N* 2 s epochs (columns of **S**), where 2*N* s is the duration of the EEG recording. The feature vector at epoch *n* is given by $x_{n}^{\left(SDB\right)}=s_{n}={\left[s_{n,0},s_{n,1},\ldots ,s_{n,100}\right]}^{T}$, which is a vector of estimates of spectral power between 0 and 50 Hz in 0.5 Hz bands.
- **Bandwise power in canonical spectral ranges (X^(*BWP*)^):** EEG activity in canonical frequency ranges relates to unconsciousness [2]. Power in these bands was estimated from the multitaper power spectral estimate and was given to the ensuing classification model in dB. The bands used were: slow (0-1 Hz), delta (1-4 Hz), theta (4-8 Hz), alpha (8-13 Hz), beta (13-25 Hz), and gamma (25-50 Hz), as delineated in [2]. Thus, the X^(*BWP*)^ feature set $B\in R^{6\times N}$ has feature vector at epoch *n* of the form $x_{n}^{\left(BWP\right)}=b_{n}={\left[b_{n,s},b_{n,\delta },b_{n,\theta },b_{n,\alpha },b_{n,\beta },b_{n,\gamma }\right]}^{T}$.
- **Principal components of the multitaper spectrogram (X^(*PCA*)^):** PCA is a technique for linearly transforming observations of multiple correlated variables into a collection of linearly uncorrelated variables of decreasing variance, enabling the dimensionality of the data to be reduced while capturing the variation within the dataset. In the present context, the observations are considered to be the 100-dimensional vectors corresponding to the mean-centered multitapered power spectral estimate in a given time window, and each principal component can be viewed as a set of weights over the power in various frequency bins. Formally, let $S\in R^{100\times N}$ be a matrix whose columns correspond to the *N* 2 s windows (comprised of *all* subjects included in training) and whose columns correspond to the 100 frequency bins from 0 Hz to 50 Hz. The *i*^*th*^ principal component $w_{i}\in R^{100}$ is then given by the eigenvector corresponding with the *i*^*th*^ largest eigenvalue of the matrix **S** **S**^*T*^ and the *i*^*th*^ principal component *score* for a window $s_{n}\in R^{100}$ (*n* = 1, …,*N*) is computed as $p_{n,i}=s_{n}^{T}w_{i}$. Given that this procedure is unsupervised (i.e. performed independently of the consciousness labels), there is no guarantee that the principal component scores will enable effective discrimination between the conscious/unconscious classes. By plotting the principal components as a function of frequency, it is evident that the first three principal components correspond with clinically relevant features and capture 92.1% of the total variance (see S2 Fig). As such, the PCA features for a given time *n* are given by $x_{n}^{\left(PCA\right)}=p_{n}={\left[p_{n,1},p_{n,2},p_{n,3}\right]}^{T}$. For testing and generalization phases, the principal components **w**_*i*_ from training were used to compute the principal component scores corresponding to a new 2 s window. PCA was performed using scikit-learn [37].
- **The linear discriminant of the multitaper spectrogram (X^(*LDA*)^):** Linear Discriminant Analysis (LDA) is a supervised learning dimensionality reduction technique that removes redundant or dependent elements from higher dimensional space by transforming the features to lower dimensional space. LDA can be viewed as a supervised analogue of PCA, where rather than simply maximizing the variance of the variables in the transformed space, it seeks to maximizes the ratio of between-class to within-class separability [38]. This yields a *linear discriminant* which maximally separates the two classes. Specifically, let $S^{\left(0\right)}\in R^{100\times N_{0}}$ and $S^{\left(1\right)}\in R^{100\times N_{1}}$ be the subsets of the complete data **S** corresponding to 2 s windows of consciousness and unconsciousness, respectively, with *N*_0_ + *N*_1_ = *N*. For *j* ∈ {0,1}, let ***μ***_*j*_ be the sample mean of **S**^(*j*)^ and define the *scatter matrix* as the unnormalized sample covariance matrix $M_{j}=\sum_{n\in N_{j}}\left(s_{n}^{\left(j\right)}-\mu_{j}\right){\left(s_{n}^{\left(j\right)}-\mu_{j}\right)}^{T}$. *Within-class* scatter matrix is defined as **M**_*W*_ = **M**_0_ + **M**_1_ and the *between-class* scatter matrix was defined as **M**_*B*_ = (***μ***_0_ − *μ*_1_)(***μ***_0_ − ***μ***_1_)^*T*^, with the goal of projecting the data into a space where there is a large variability between classes but a small variability within a each class. Thus, the linear discriminant $v^{*}\in R^{100}$ is found as the solution to:
  $$\begin{array}{c} \underset{v}{\mathrm{argmax}}\frac{v^{T}M_{B}v}{v^{T}M_{W}v} \end{array}$$ (1)
  Finally, the resultant feature from a 2 s window is found by projecting the multitapered power spectral estimate onto the linear discriminant: $x_{n}^{\left(LDA\right)}=l_{n}=s_{n}^{T}v^{*}$. LDA was performed using scikit-learn [37].
- **Convolutional neural network (X^(*CNN*)^) features:** Convolutional neural networks (CNNs) are deep neural networks that use convolution operations to compute spatial features in an image. In this case, spatial convolutions of the multitaper spectrogram correspond to time/frequency relationships within the EEG recording. A previously described neural network architecture called MobileNet was used for this purpose because it is a relatively low-capacity network [39]. This network architecture is built-in to deep learning libraries (https://www.tensorflow.org/api_docs/python/tf/keras/applications/MobileNet). We replaced the final fully connected and 1000-output softmax layers with a single 2-output softmax layer. Because of the limited amount of EEG training data, image features were those learned for photographic image recognition in the ImageNet challenge [40], and the network was trained to associate these features with conscious state in a process called transfer learning. Transfer learning has previously been used to train CNN models for image recognition tasks in other medical fields such as radiology [41–43] and pathology [44]. CNN model inference was performed using the keras library with tensorflow backend [45].
  The CNN input was the multitapered power spectral estimate for the preceding 30 s with 2 s steps between windows (28 s overlap). Pixel intensities for an entire case were normalized to values between 0 and 1. The CNN mapped each $R^{15\times 100}$ 30 s spectrogram window into $v_{n}\in R^{1280}$ features, resulting in the full CNN feature matrix $V\in R^{1280\times (N-28)}$. A PCA was performed on the CNN feature matrix **V** in an analogous manner to above, reducing the CNN feature matrix to its first ten PC scores. This yielded the CNN features $x_{n}^{\left(CNN\right)}\in R^{10}$ at each of (*N* − 28) epochs.

#### Time series analysis with Hidden Markov modeling

HMMs were used to incorporate temporal patterns into model predictions. A HMM is a state-space model that has been used to describe time series data in a wide variety of fields. It assumes an *M*-state system has {*q*_0_, *q*_1_, …*q*_*M*−1_} discrete latent states which evolve over time, driven by a first-order, ergodic Markov chain resulting in a sequence of states *Z* = (*z*_0_, *z*_1_, …,*z*_*N*_). Observations of the system (in our case, the EEG features defined previously) are distributed according to state-specific Gaussian emission distributions **B** = {**b**_*m*_} where $b_{m}=N\left(\mu_{m},\Sigma_{m}\right)$ for each state *q*_*m*_ with mean ***μ***_*m*_ and covariance ***Σ***_*m*_. In our application, ***Σ***_*m*_ was constrained to be diagonal.

The Markov chain transition matrix is *A* = {*a*_*ij*_} where *a*_*ij*_ = *P*(*z*_*n*+1_ = *q*_*j*_|*z*_*n*_ = *q*_*i*_). The initial state of the system is drawn from the discrete initial state distribution *π*. The entire HMM is fully parameterized by ***λ*** = (*A*, **B**, *π*). In the model system, the state of the system at each discrete time *n* is based on the Markov chain transition probabilities and an observed feature is generated according to current state *z*_*n*_ resulting in a sequence of observations **X** = (**x**_0_, **x**_1_, …,**x**_*N*_). [46]

Each HMM was trained with the Baum-Welch algorithm using one of the five previously-described features (computed from healthy volunteer data) as the observations of the model system. For prediction of latent state, only the HMM forward algorithm was used so the classification models are applicable for future real-time implementation. The normalized forward probability is defined as *α*(*q*_*i*_) = *P*(*z*_*n*_ = *q*_*i*_|*x*_0_, …,*x*_*n*_, *λ*). One of three time series treatments was applied to each feature: a 2 state HMM, a 6 state HMM, or no time series treatment. A 2-state HMM was chosen to match the binary classification scheme, where each latent state reflects a subject state of consciousness or unconsciousness. A 6-state HMM was also used to reflect that there may exist multiple distinct EEG states which may then map to conscious or unconscious subject. Despite using a binary classification scheme, intermediate states may arise during the transition from consciousness to unconsciousness. Purdon *et al.* [27] showed that unique spectral EEG features appear before complete loss of consciousness and again after returning to consciousness, but before the return of normal behavior. Six states were chosen based on the six increasing steps of target effect site concentration in the healthy volunteer cohort (Fig 4). AIC was calculated for a range of 2-30 state numbers, but no minimum was found.

Thus, for each feature vector $x_{n}^{\left(f\right)}$ at 2 s epoch *n* for *f* ∈ {*SDB*, *BWP*, *PCA*, *LDA*, *CNN*}, the 2-state HMM yielded $x_{n}^{\left(f+HMM2\right)}\in R^{2}$, and the 6-state HMM yielded $x_{n}^{\left(f+HMM6\right)}\in R^{6}$.

In some cases in the surgical cohorts, the EEG signal was temporarily interrupted. Because the HMM was used to model the temporal dynamics of the system, only continuous lengths of data were used. In surgical cohorts cases where the EEG signal did drop out, the HMM forward algorithm was re-initialized at the start of where the EEG signal came back online. The HMM models were built using the Python hmmlearn package (https://github.com/hmmlearn/hmmlearn), modified to use the output of the forward algorithm alone for prediction.

#### Classification with logistic regression

Logistic regression (LR) is a standard approach to binary classification tasks [36]. For each combination of feature generation technique and time series treatment described above, a different LR model was trained. Let **x**_*n*_ = [*x*_*n*,1_, *x*_*n*,2_, …,*x*_*n*, *K*_]^*T*^ be a feature vector corresponding to any of the techniques described above, where the dimension *K* of the feature vector will be determined by the specific featurization method. For each window *n*, the feature vector is accompanied by a label *y*_*n*_ ∈ {0,1} indicating whether the window corresponds with conscious or unconscious brain activity. A LR model is parameterized by a vector $\beta \in R^{K+1}={\left[\beta_{0},\beta_{1},\ldots ,\beta_{K}\right]}^{T}$. For a given parameterization ***β***, the LR estimated probabilities of consciousness and unconsciousness for window *n* are given by:

$$\begin{array}{c} Pr\left(\text{Conscious}|x_{n};\beta \right)=\frac{\exp\beta^{T}{\tilde{x}}_{n}}{1+\exp\beta^{T}{\tilde{x}}_{n}}\;\;\;\;\;Pr\left(\text{Unconscious}|x_{n};\beta \right)=\frac{1}{1+\exp\beta^{T}{\tilde{x}}_{n}} \end{array}$$ (2)

where ${\tilde{x}}_{n}={\left[1,x_{n}^{T}\right]}^{T}$ is the feature vector with a one prepended, enabling *β*_0_ to serve as a constant offset. Thus, training a LR model entails finding the parameter set that maximizes the *ℓ*_2_-regularized log-likelihood of the labels corresponding to the training set:

$$\begin{array}{c} \hat{\beta }=\underset{\beta }{\mathrm{argmin}}\sum_{n=1}^{N}-\log Pr\left(y_{n}|x_{n};\beta \right)+\frac{1}{2}{\left|\left|\beta \right|\right|}_{2}^{2} \end{array}$$ (3)

where ${∥\beta ∥}_{2}^{2}=\beta^{T}\beta$. Given that this procedure is repeated across featurization methods, likelihood maximizing parameter set will be obtained for each (for example, ${\hat{\beta }}^{\left(SDB\right)}$ trained on **X**^(*SDB*)^). In all cases, the parameter vector was computed using scikit-learn with *ℓ*_2_ regularization and the liblinear solver [37].

### Statistical analyses

#### Cross-validation and model selection

Seven-fold leave-one-out cross-validation [36] was used to compare model performance using the seven healthy volunteers in the volunteer training cohort. PCA and LDA features were fit and each model was trained on six cases for each fold and validated on the held-out case. Model performance was compared using area under the receiver operating characteristic curve (description in ensuing section). Due to the small number of independent samples (seven patients) tests for statistical significance between models were not performed. We elected to keep three high-performing models with different features and temporal models to gain insight into how these characteristics affect model performance and interpretation.

#### Model testing on healthy volunteers and surgical patients

Classification models were evaluated on data recorded from subjects that were not used in model training: held-out healthy volunteers, patients surgery procedures using propofol, and patients undergoing surgery where sevoflurane was the primary anesthetic. For each of the three classifiers selected and each test cohort, a receiver operating characteristic (ROC) curve, the area under the ROC curve (AUC), and the classifier accuracy were computed. The ROC curve is the plot of true positive (*TP*) rate (*TPR* = *TP*/(*TP* + *FN*), where *FN* = false negative count) vs. false positive (*FP*) rate (*FPR* = *FP*/(*TN* + *FP*) where *TN* = true negative count) while varying the threshold between positive and negative. An AUC of 0.5 is equivalent to random labeling, and an AUC of 1.0 is perfect performance. Accuracy, the fraction of the time the classifier is correct: *ACC* = (*TP* + *TN*)/(*TP* + *FP* + *TN* + *FN*), was also calculated. Accuracy was calculated for thresholds of 0.5 (default threshold) or personalized thresholds, selected by computing optimal threshold *t*_*opt*_:

$$t_{opt}=\arg\max_{t}\left(1-FPR\left(t\right)\right)+TPR\left(t\right)$$

where *t* is a threshold between 0 and 1. AUC is generally a more valuable statistic because it is balanced by the incidence of positives and negatives. AUC was also computed by case to determine if errors predominantly occurred for specific patients, or if they were distributed throughout the data.

## Supporting information

S1 Fig Propofol surgical patient for which classifier performance is worst for every classifier. This surgery was a 21-year-old woman undergoing extracorporeal shock wave lithotripsy. Propofol was repeatedly bolused during the early portion of the procedure. The classifier predicts consciousness until the penultimate bolus, after which the classifier predicts a rapid shift to a deeply sedated state. We interpreted this as the clinician seeking a deeper level of sedation due to stimuli during the procedure. Rather than performing poorly, our classifier is apparently capturing this phenomenon accurately.
(TIF)

S2 Fig Visualization of PCA and LDA features. (A) Multitaper spectrogram for a test healthy volunteer, used to visualize how PCA and LDA transform a spectrogram S. Loss and return of consciousness (as defined in Methods) are indicated by white vertical lines. (B) Fraction of data variance explained by each of the first ten principal components. All PCs past PC3 explained <2% of data variance and were thus excluded. (C) Clinical interpretation of the first three PCs corresponds well with understanding of how the multitaper spectrogram evolves during propofol anesthesia. PC1 is the overall power. PC2 is predominantly the gamma power minus the slow-delta, theta, and alpha power (all known to be higher during unconsciousness). PC3 may be thought of as the slow-delta power (high during unconsciousness) minus the beta power (high during the transition between consciousness and unconsciousness). (D) Plotting the dynamics of the PC score during the subject in A shows interpretation of PC scores. PC1 increases slightly during unconsciousness, but is highly noisy. PC2 is high during consciousness and low during unconsciousness. PC3 is high during consciousness, low during the transition from conscious to unconscious, and high during the transition between states. (E) Linear discriminant vector. Although vector values are less readily interpretable, trends similar to PC2 may be seen: predominantly negative values for slow-delta and alpha bands, and positive values for high-frequency gamma. (F) Likewise, the overall temporal dynamics of the LD score is visually similar to the trend for PC2 for this example subject.
(TIF)

S3 Fig Timing of classifier shows real-time capability. We computed the time it took to perform classification for each 2 s window of raw EEG data for an example healthy volunteer case and an example case from the surgical cohort. We found that computation time for classification was <0.1s for all windows, and thus the algorithm may be run in real time. Computation was performed on MacBook Pro using a 2.4 GHz Quad-Core Intel® Core i5 with 16 GB RAM.
(TIF)

S4 Fig Classification model performance on a healthy volunteer subject with burst suppression classification results circled in red. Although this region was predominantly labeled unconscious, some 2 s windows were labeled conscious. A three-class classification model might add burst suppression as an additional undesirable state during surgery where the patient is at a different level of unconsciousness closely resembling coma.
(TIF)
