# Supplementary figures and images for: Machine learning of EEG spectra classifies unconsciousness during GABAergic anesthesia
Source: PLoS One. 2021 May 6;16(5):e0246165. doi: 10.1371/journal.pone.0246165 (PMC8101756; doi:10.1371/journal.pone.0246165)

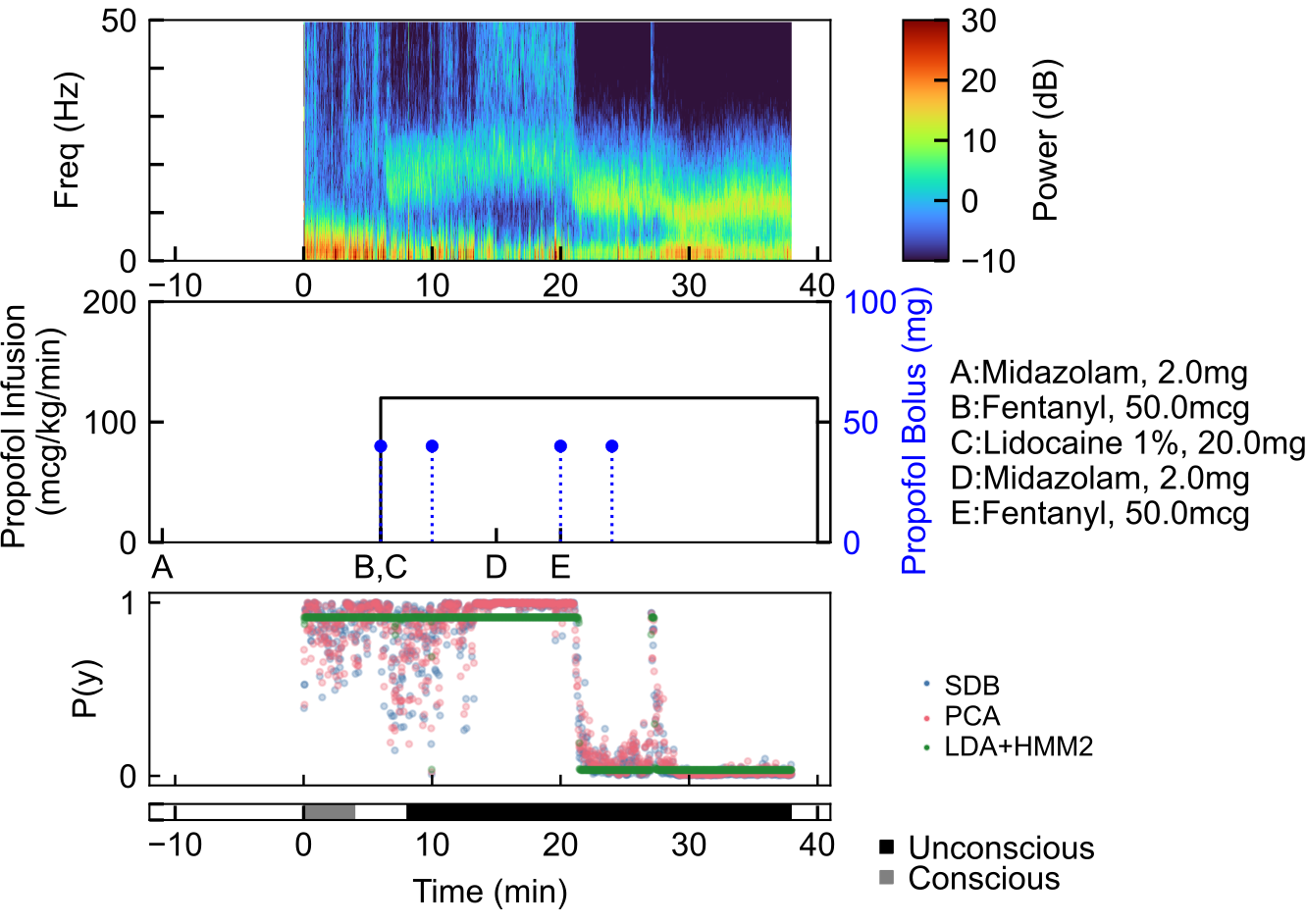

Supplement: S1 Fig — This surgery was a 21-year-old woman undergoing extracorporeal shock wave lithotripsy. Propofol was repeatedly bolused during the early portion of the procedure. The classifier predicts consciousness until the penultimate bolus, after which the classifier predicts a rapid shift to a deeply sedated state. We interpreted this as the clinician seeking a deeper level of sedation due to stimuli during the procedure. Rather than performing poorly, our classifier is apparently capturing this phenomenon accurately. (TIF) [file pone.0246165.s001.tif]

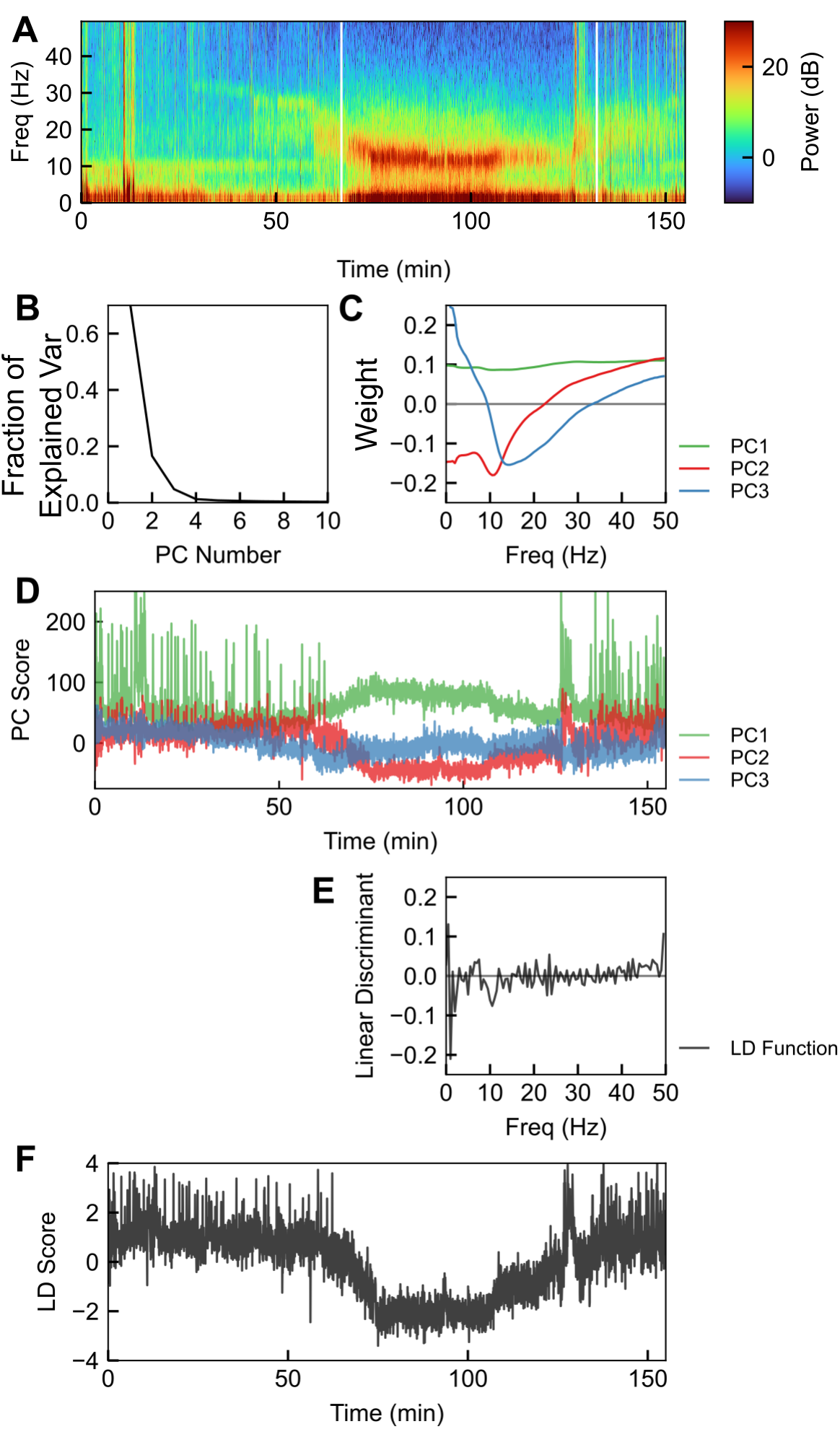

Supplement: S2 Fig — (A) Multitaper spectrogram for a test healthy volunteer, used to visualize how PCA and LDA transform a spectrogram S. Loss and return of consciousness (as defined in Methods) are indicated by white vertical lines. (B) Fraction of data variance explained by each of the first ten principal components. All PCs past PC3 explained <2% of data variance and were thus excluded. (C) Clinical interpretation of the first three PCs corresponds well with understanding of how the multitaper spectrogram evolves during propofol anesthesia. PC1 is the overall power. PC2 is predominantly the gamma power minus the slow-delta, theta, and alpha power (all known to be higher during unconsciousness). PC3 may be thought of as the slow-delta power (high during unconsciousness) minus the beta power (high during the transition between consciousness and unconsciousness). (D) Plotting the dynamics of the PC score during the subject in A shows interpretation of PC scores. PC1 increases slightly during unconsciousness, but is highly noisy. PC2 is high during consciousness and low during unconsciousness. PC3 is high during consciousness, low during the transition from conscious to unconscious, and high during the transition between states. (E) Linear discriminant vector. Although vector values are less readily interpretable, trends similar to PC2 may be seen: predominantly negative values for slow-delta and alpha bands, and positive values for high-frequency gamma. (F) Likewise, the overall temporal dynamics of the LD score is visually similar to the trend for PC2 for this example subject. (TIF) [file pone.0246165.s002.tif]

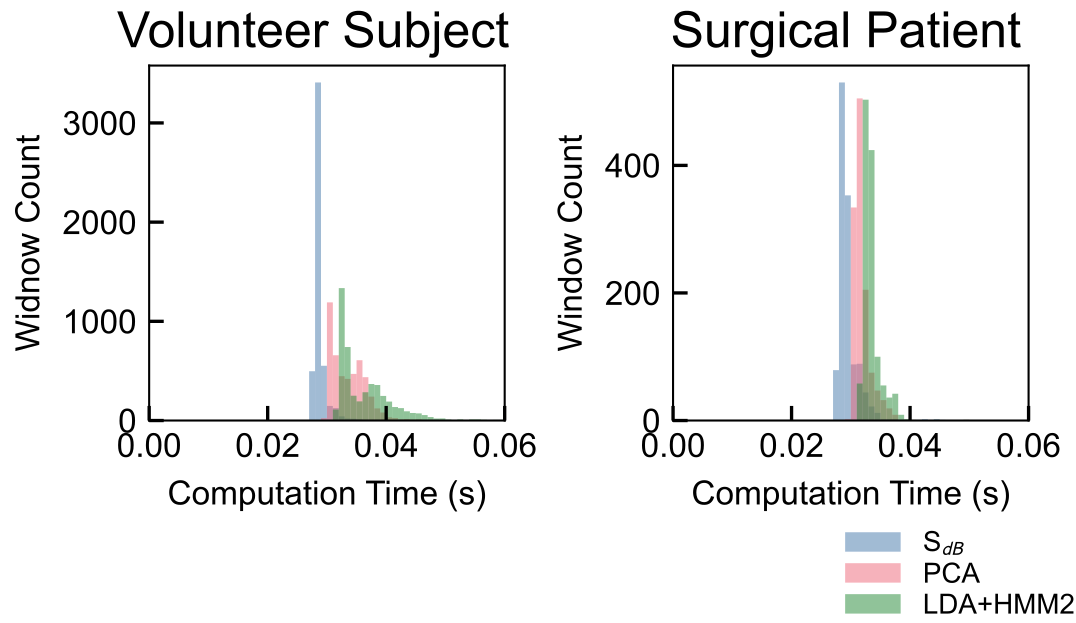

Supplement: S3 Fig — We computed the time it took to perform classification for each 2 s window of raw EEG data for an example healthy volunteer case and an example case from the surgical cohort. We found that computation time for classification was <0.1s for all windows, and thus the algorithm may be run in real time. Computation was performed on MacBook Pro using a 2.4 GHz Quad-Core Intel® Core i5 with 16 GB RAM. (TIF) [file pone.0246165.s003.tif]

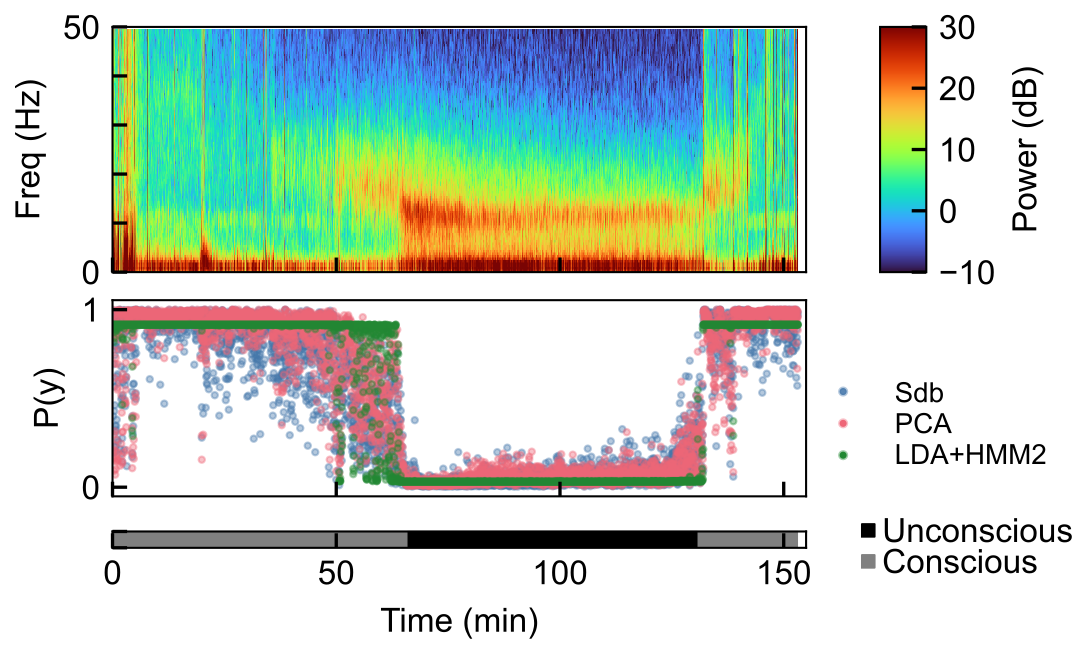

Supplement: S4 Fig — Although this region was predominantly labeled unconscious, some 2 s windows were labeled conscious. A three-class classification model might add burst suppression as an additional undesirable state during surgery where the patient is at a different level of unconsciousness closely resembling coma. (TIF) [file pone.0246165.s004.tif]
